# Skim-Sequencing Based Genotyping Reveals Genetic Divergence of the Wild and Domesticated Population of Black Tiger Shrimp (*Penaeus monodon*) in the Indo-Pacific Region

**DOI:** 10.3390/biology9090277

**Published:** 2020-09-07

**Authors:** Li Lian Wong, Zulaikha Mat Deris, Yoji Igarashi, Songqian Huang, Shuichi Asakawa, Qasim Ayub, Shu Yong Lim, Mhd Ikhwanuddin, Shumpei Iehata, Kazutoshi Okamoto, Mariom, Md Asaduzzaman

**Affiliations:** 1 Institute of Marine Biotechnology, Universiti Malaysia Terengganu, Kuala-Terengganu 21030, Terengganu, Malaysia; zulaikhamd27@gmail.com; 2 Graduate School of Bioresources, Mie University, Kurimamachiya 1577, Tsu, Mie 514-8507, Japan; igarashi@bio.mie-u.ac.jp; 3 Department of Aquatic Bioscience, The University of Tokyo, 1-1-1 Yayoi, Bunkyo-ku, Tokyo 113-8657, Japan; huangsongqian0115@gmail.com (S.H.); asakawa@mail.ecc.u-tokyo.ac.jp (S.A.); 4 School of Science, Monash University Malaysia Genomics Facility, Bandar Sunway 47500, Selangor, Malaysia; qasim.ayub@monash.edu (Q.A.); lim.shuyong@monash.edu (S.Y.L.); 5 Institute of Tropical Aquaculture and Fisheries, Universiti Malaysia Terengganu, Kuala-Terengganu 21030, Terengganu, Malaysia; ikhwanuddin@umt.edu.my; 6 Faculty of Fisheries and Food Science, Universiti Malaysia Terengganu, Kuala-Terengganu 21030, Terengganu, Malaysia; shumpei@umt.edu.my; 7 Shizuoka Prefectural Research Institute of Fishery and Ocean, Yaizu-shi, Shizuoka 425-0032, Japan; kazutoshi1_okamoto@pref.shizuoka.lg.jp; 8 Department of Fisheries Biology and Genetics, Faculty of Fisheries, Bangladesh Agricultural University, Mymensingh 2202, Bangladesh; mariom.bau@gmail.com; 9 Department of Marine Bioresource Science, Faculty of Fisheries, Chattogram Veterinary and Animal Sciences University, Khulshi 4225, Chattogram, Bangladesh

**Keywords:** low coverage sequencing, population structure, genetic improvement, outlier approach, domesticated population, *Penaeus monodon*

## Abstract

The domestication of a wild-caught aquatic animal is an evolutionary process, which results in genetic discrimination at the genomic level in response to strong artificial selection. Although black tiger shrimp (*Penaeus monodon*) is one of the most commercially important aquaculture species, a systematic assessment of genetic divergence and structure of wild-caught and domesticated broodstock populations of the species is yet to be documented. Therefore, we used skim sequencing (SkimSeq) based genotyping approach to investigate the genetic structure of 50 broodstock individuals of *P. monodon* species, collected from five sampling sites (*n* = 10 in each site) across their distribution in Indo-Pacific regions. The wild-caught *P. monodon* broodstock population were collected from Malaysia (MS) and Japan (MJ), while domesticated broodstock populations were collected from Madagascar (MMD), Hawaii, HI, USA (MMO), and Thailand (MT). After various filtering process, a total of 194,259 single nucleotide polymorphism (SNP) loci were identified, in which 4983 SNP loci were identified as putatively adaptive by the pcadapt approach. In both datasets, pairwise F_ST_ estimates high genetic divergence between wild and domesticated broodstock populations. Consistently, different spatial clustering analyses in both datasets categorized divergent genetic structure into two clusters: (1) wild-caught populations (MS and MJ), and (2) domesticated populations (MMD, MMO and MT). Among 4983 putatively adaptive SNP loci, only 50 loci were observed to be in the coding region. The gene ontology (GO) and Kyoto Encyclopedia of Genes and Genomes (KEGG) analyses suggested that non-synonymous mutated genes might be associated with the energy production, metabolic functions, respiration regulation and developmental rates, which likely act to promote adaptation to the strong artificial selection during the domestication process. This study has demonstrated the applicability of SkimSeq in a highly duplicated genome of *P. monodon* specifically, across a range of genetic backgrounds and geographical distributions, and would be useful for future genetic improvement program of this species in aquaculture.

## 1. Introduction

The world production of inland aquaculture reached 51.3 million tonnes in 2018, with their dominant production of 97.2% comprising of finfish, while mariculture produced 30.8 million tonnes, with 56.2% represented by mollusc [1]. Marine shrimp industries yielded almost 4 million tonnes in the same year, playing the role as the major supply of shrimps in the global market [2]. While disease outbreak and price fluctuations in the global trade have highly impacted the production and socio-economic development in many countries, the surging prices and shortage of good quality shrimp broodstocks have further impeded the shrimp industry [1,3]. In fact, shrimp farming is heavily dependent on wild-caught broodstocks or domesticated broodstocks with reduced quality, due to repeated spawning programs [4,5,6]. To ensure sustainable seed supply, standardized benchmarks for quality and quantity assessment of broodstocks are essential [7].

Population structure is the organization of genetic diversity, and it is influenced by multiple evolutionary process, such as genetic drift, mutation, gene flow, natural selection and demographic history [8,9]. It is estimated by parameters like genetic differentiation, variant alleles frequencies, population size and population dynamic [10,11,12,13]. Low number of breeders and inadvertent or mass selection of prospective shrimp broodstocks can result in rapid reduction of genetic diversity [14,15,16,17,18,19]. The loss of genetic variation, reduction in effective population size and accumulation of inbreeding effects over several generations of selective breeding can compromise the effectiveness of genetic improvement programs [20,21,22]. Although the magnitude of inbreeding effects are variable between different traits, genetic structure of a population and environmental interactions, the overall mean phenotypic value of traits associated with reproductive fitness and physiological efficiency is often reduced [23,24,25]. Therefore, adequate levels of genetic variation are vital for the maintenance of the gene pool in cultured shrimp with minimal effects of inbreeding depression and enhanced capacity in responding to environmental changes [26,27]. Long-term crossbreeding programs may help to achieve a balance between continuous genetic gains and reduced risk of inbreeding depression [6,28].

*Penaeus monodon*, commonly known as black tiger shrimp is widely distributed across the Indo-Pacific region, and is one of the most commercially important aquaculture species [29,30]. To date, population structure studies of *P. monodon* have only been limited to several traditional markers such as mtDNA RFLP [29], allozymes [31], microsatellite [32,33] and Sanger sequencing of various genes including mtCR (mtDNA control region) [34,35], mitochondria DNA [36] and elongation factor-1a [37], while next generation sequencing (NGS) approaches are yet to be explored for this species in this aspect. In fact, NGS have been well proven in revealing fine-scale population structure and phylogeographic divergence in numerous aquatic species [38,39,40,41,42,43,44].

Skim sequencing (SkimSeq) is one of the less complex NGS methods, which uses low coverage (1–10X) whole genome sequencing of multiple individuals for high resolution genotyping [45,46,47,48]. SkimSeq is less laborious with fewer complex steps, is unbiased towards specific alleles, is capable of SNPs detection, which enables informative sampling and validation of the genome [49,50,51,52,53]. This genotyping by sequencing approach is useful in population study with unknown parental genome information to generate detailed diversity analysis and marker-assisted selections [45,53]. 

Given that the SkimSeq is known as low coverage genome sequencing approach [38], and so far have only been applied in plant genomics research [45,46,47,48], we have chosen this technique to generate high resolution sequence dataset, as reported in previous studies, for the first aquatic invertebrate with small samples size. To explore the genetic diversity, population structure and discover the novel molecular markers of *P. monodon* from different origins (wild vs. domesticated), we genetically assayed 50 individuals with the SkimSeq approach using the short read Illumina sequencing platform. This study aimed to: (i) investigate the genetic structure of wild and domesticated populations, (ii) quantify the genetic differentiation between populations and (iii) examine the presence of putative loci causing the genetic variation. The genomic analyses and genetic resource acquired from this study will be useful to support future genetic improvements in *P. monodon* culture and brood-stock selection activities.

## 2. Materials and Methods

### 2.1. Sample Collection

**Figure 1:**
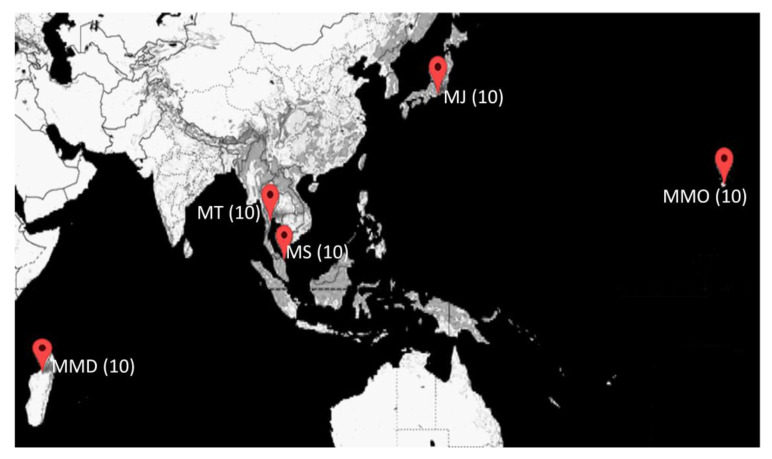
Sampling sites of five broodstock populations of *Penaeus monodon* in the Indo-Pacific region. MMD (Madagascar), MT (Thailand) and MMO (Hawaii, HI, USA) represent domesticated populations, while MS (Malaysia) and MJ (Japan) are wild-caught populations. Values in parentheses denote the sample size for each population.

A total of 50 *P. monodon* individuals consisting of both domesticated and wild-caught broodstocks were collected from five sampling sites (10 individuals from each site and of same family) across Indo-Pacific regions (Figure 1), and the sampling details are listed in Table 1. Among the five locations, *P. monodon* broodstock populations from MMD (Madagascar), MT (Thailand) and MMO (Hawaii, HI, USA) were obtained from domesticated shrimp farms, while MS (Malaysia) and MJ (Japan) were caught from the wild. The muscle tissues were extracted from broodstock individuals and preserved in 99.5% *v*/*v* ethanol and stored at −20 °C until DNA extraction. All samples were collected in accordance with the animal care and tissue collection protocol as approved by the Universiti Malaysia Terengganu’s Animal Care and Biosafety Committee.

### 2.2. DNA Extraction and Library Preparation

Genomic DNA of *P. monodon* broodstocks were isolated from the muscle tissues using Wizard Genomic DNA Purification Kit following the manufacturer’s protocols (Promega, San Luis Obispo, CA, USA). The concentration and purity of the extracted genomic DNA were quantified based on A260/280 nm ratio using BioDrop (BioDrop, Cambridge, UK). DNA quantifications were conducted using real-time PCR fluorescence measurements of double stranded DNA [54] and the Quant-it kit (Life Technologies, Foster City, CA, USA). Genomic DNA was fragmented into the insert size of 350 bp using with TruSeq^®^ DNA PCR-Free Library Prep Kit (Illumina Inc., San Diego, CA, USA) and Covaris M220 (Covaris Inc., Woburn, MA, USA), following the kits’ protocol at Monash University Malaysia Genomics Facility (Selangor, Malaysia). Accurate quantification and quality checking of the DNA libraries were conducted using the KAPA Library Quantification Kit (Roche Sequencing and Life Science, Indianapolis, IN, USA), while qualitative estimation of the libraries was performed by Agilent Technologies 2100 Bioanalyzer (Agilent Technologies Inc., Santa Clara, CA, USA). Detailed QC parameters, including library cluster density, library complexity, percent duplication, GC bias, and index representation were generated on the MiSeq system (Illumina Inc., Foster City, CA, USA) to ensure a uniform concentration of all samples prior to SkimSeq. The pooled library was denatured based on the Illumina NextSeq denaturation guideline and paired-end sequencing was carried out using NextSeq 500/550 High Output v2 300 cycles kit on a NextSeq 500 (Illumina Inc., Foster City, CA, USA).

### 2.3. Sequence Assembly, Filtering and SNPs Discovery

From the Illumina platform, 151 base-pair paired-end (2 × 151) sequencing reads were obtained in FASTQ format. Sequencing reads matching to PhiX DNA sequences were first removed by aligning reads against the PhiX DNA sequence using Bowtie 2 software version 2.2.3 (http://bowtie.cbcb.umd.edu) [55,56]. The cleaned reads were then subjected to Illumina sequencing adapter trimming and base quality (Q ≥ 20) trimming using PEAT software version 1.2.4 (http://jhhung.github.io/PEAT) [57], to ensure that only good quality bases derived from the sample were further analyzed. Trimmed reads of less than 36 bp were also discarded using Trimmomatic software version 0.36 (http://www.usadellab.org/cms/index.php?page=trimmomatic) [58]. The quality trimming and filtering analyses of the whole genome SkimSeq data revealed that each of the 50 individuals of *P. monodon* had about 30% low-quality reads, which were discarded before *de novo* genome assembly (Table S1). In the present study, the good quality reads of all samples were assembled collectively using SOAPdenovo software version 2-r240 (ftp://public.genomics.org.cn/BGI/SOAPdenovo2) [59] into a set of scaffolds. All samples (except MJ10) had greater than 91% mapping rate to the assembled scaffold, indicating that the assembled scaffold is able to comprehensively represent all samples (Table S2). The calculated coverage of SkimSeq data in different samples was found to vary from 1.3 to 1.8X. Subsequently, BLAST+ version 2.2.31 (ftp://ftp.ncbi.nlm.nih.gov/blast/executables/blast+/LATEST) [60] was used to identify the coding protein sequences from the order of Decapoda and the full SwissProt database [61] to the assembled scaffold sequences. The good quality sequencing reads were aligned to the assembled scaffold sequences using BWA version 0.7.12-r1039 (http://maq.sourceforge.net) [62]. The reads alignments were sorted, and potential PCR duplicate reads were identified and marked using Picard Tools version 2.9.0. The reads alignments were analyzed to identify variants using FreeBayes version 1.2.0-2-g29c4002 [63] to find small polymorphisms, specifically SNPs (single-nucleotide polymorphisms), indels (insertions and deletions), MNPs (multi-nucleotide polymorphisms) and complex events (composite insertion and substitution events) smaller than the length of a short-read sequencing alignment. The outputs includes a total number of 17,226,908 raw variants loci in the VCF file format. The identified variants coordinates were compared to the gene coordinates obtained from genome annotation using vcfanno software version 0.2.9 (https://github.com/brentp/vcfanno) [64]. If a variant fell within the region for a protein-genome match, the information of the protein name was transferred to the variants as an annotation.

SNP profiles were analyzed and visualized using SNPRelate version 1.16.0 [65], to exclude outlier samples with inconsistent genetic profiles. Likewise, samples containing a completeness of data of less than 80% among the remaining loci, a minimum quality score with minor allele frequency below threshold, and a mean depth per genotype lower than 20 were removed from the dataset. Out of the 50 *P. monodon*, 5 individual sequences (MJ10, MS9, MMO1, MMO7 and MMO16) were removed from the dataset due to the inconsistent SNP profiles and/or greater than 20% missing genotypes, while the remaining 45 samples were used for all downstream analyses. Furthermore, variants filtering was conducted using VCFtools software version 0.1.16 (https://vcftools.github.io/) [66] and BCFtools software version 1.9 (http://samtools.github.io/bcftools/bcftools.html). At first, the variants filtering steps included the removal of complex indels, SNPs with more than two alleles, and composite insertion and substitution events. Further filtering steps included the removal of sites with less than 5% overall minor allele frequency, missing genotypes in >90% of the samples in any population, and SNP sites with genotypes not in Hardy-Weinberg equilibrium in any population (PHWE < 0.001). After all filtering steps, a total of 194,259 individual SNP loci remained in the dataset. To detect putatively adaptive SNP loci among different wild and domesticated broodstock populations of *P. monodon*, we identified outlier SNPs from the 194,259 filtered individual SNP loci using pcadapt version 3.0.2 [67,68]. The “pcadapt” approach performs a principal component analysis and computes the *p*-values of each locus to detect adaptive loci. Default parameters were used for pcadapt analysis, and the “number_of_samples” parameter was set to 5 (a number equal to the sampled collections). The false discovery rate (FDR) threshold value set to 0.05, to control the false positive. Finally, an overall SNP loci dataset and putatively adaptive SNP loci dataset were created and used for all down-stream spatial clustering analyses.

### 2.4. Power Analysis

A power analysis using POWSIM v. 4.1 [69] was carried out to determine the power of all SNP loci and putatively adaptive SNP loci datasets derived from the SkimSeq approach. This program evaluates the statistical power of genetic homogeneity in individual species and allows the user to adjust a number of user-defined parameters. To calculate the power of our sampling design, the number of subpopulations was set to five (equals to the number of our collection sites), with 10 samples per subpopulation (number of the collected individuals per sampling site) for both all of the SNP loci and the putatively adaptive SNP loci datasets. The effective population size of the subpopulations were set to 1000, 2000 and 3000, and generation time (t) was adjusted to assess power at multiple F_ST_ values (10 and 20 generations), as described previously [39]. As F_ST_ in POWSIM assumes the independence of the subpopulations, power was expressed as the proportion of significant outcomes for 1000 alterations per batch and a statistically significant test (*p* < 0.05).

### 2.5. Genetic Variation Analysis

The filtered data was imported as a genind object into R and down-stream spatial clustering analyses were largely conducted using the adegenet v2.0.1 R package (http://cran.r-project.org/mirrors.html) [70]. The GenoDive version 3.0 (Universiteit van Amsterdam, Amsterdam, The Netherlands) was used to conduct an analysis of molecular variance (AMOVA) on both datasets [71]. The GenoDive was also used for significance testing of pairwise F_ST_ to determine the genetic differences between collection sites for the all SNP loci and outlier datasets using the default settings, with the samples grouped by collection sites. Clustering analysis, discriminant analysis of the principal component (DAPC), was conducted using the adegenet R package (Universite’ de Lyon, UMR 5558, Lyon, France) on all SNP loci dataset and outlier datasets. Neighbor-joining trees were generated using all the SNP loci dataset and outlier datasets using Nei’s genetic distance method. The Bayesian clustering method, implemented in the STRUCTURE software v. 2.3.4 (Stanford University, Stanford, CA, USA), was used to genetically assign individuals to clusters [72]. Simulations were run for 100,000 steps, following a burn-in period of 100,000 steps, considering values of K (number of clusters) from one to 15, with 10 replications for each value of K. The analysis was performed using an admixture, correlated allele frequencies, and no prior information on the sampling location or morphological species. For each individual, the program identifies the fraction of the genome that belongs to each one of the clusters. The rate of change in the log likelihood between successive K values was also estimated [73]. The calculations were performed using STRUCTURE HARVESTER [74]. The clusters of the estimated population structure were visualized using CLUMPAK [75].

### 2.6. GO and KEGG Enrichment Analysis of Putatively Adaptive SNP Loci

To identify the genes encoded within the adaptive SNP loci, a homology search program was applied for each putatively adaptive SNP locus with the genome sequences available for the *L. vannamei* in the NCBI database. For each detected 4983 SNP loci, flanking region of upstream and downstream 100 bp was extracted from the reference nucleotide sequence. Homology search between this extracted sequence set and CDS amino acid sequences of *L. vannamei* was performed under the homology threshold of e-value = 1 × 10^−5^. Homology search program of the SNP flanking sequences showed that the total of 50 coding regions containing nonsynonymous mutations were encoded by the putatively adaptive SNP loci. To know the functional distribution of the genes encoded within the adaptive SNP loci, gene ontology (GO) and Kyoto Encyclopedia of Genes and Genomes (KEGG) pathway enrichment analysis were performed using KOBAS 3.0 software (http://kobas.cbi.pku.edu.cn/kobas3) and visualized with R [76]. The hypergeometric test was used to identify the significant GO and KEGG pathways (*p* < 0.05).

### 2.7. Data Accessibility

Our raw data for 50 individuals were submitted to the DDBJ (https://ddbj.nig.ac.jp), with the DRA accession number: DRA010601. The data will be publicly available from 10 September 2020.

## 3. Results

### 3.1. Genome Assembly, Annotation and Quality Filtering of SNP Loci

The de novo assembley of good quality data generated a total of 6,425,442 scaffolds with the largest and smallest scaffold size of 15,300 bp and 100 bp, respectively, and totaling 1,531,786,734 base pairs in length (Table 2). Genome annotation analyses displayed that 440,707 scaffolds matched with at least one protein sequence, and 107,063 Decapoda and 42,351 SwissPort protein sequences were matched to the scaffold (Table 2). A total of 17,226,908 variant sites were identified across 3,651,235 scaffolds. Among 17,226,908 variant sites, 1,347,070 were annotated with sequence similarity to Decapoda or SwissProt protein sequences (Table 2). 

**Figure 2:**
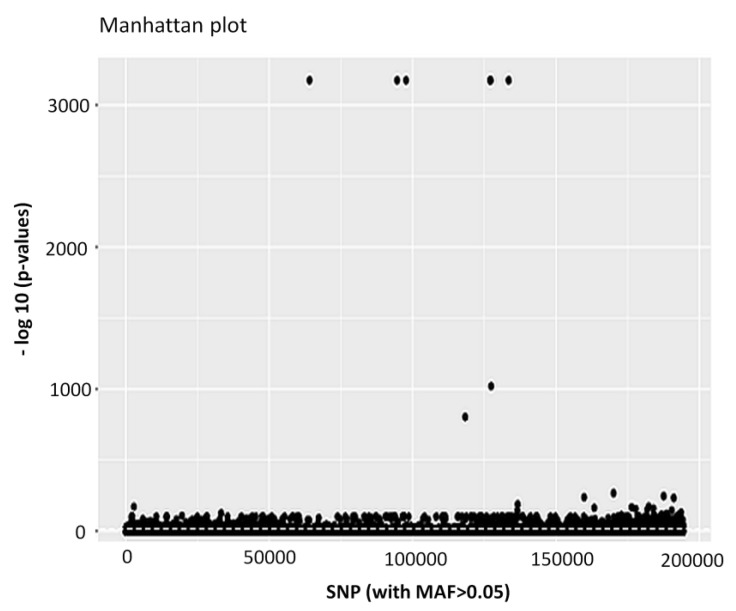
Separation of the putatively adaptive panels of single nucleotide polymorphisms (SNPs) loci, based on the pcadapt approaches. Among the 194,259 SNP loci, the pcadapt approach detected 4983 SNPs, as putative adaptive loci (above the dotted line).

Among the 17,226,908 variant sites, 10,212,187 SNP loci were retained after removing indels, MNPs and SNP sites with less than 5% minor allele frequency (Table 3). After all of the filtering steps were complete, a total of 194,259 individual SNP loci remained in the dataset. Out of the 194,259 polymorphic SNPs loci, 4983 SNP loci were identified as outliers and putatively under positive selection by the pcadapt approach (Table 3, Figure 2).

### 3.2. Power Analysis

In all of the SNP loci dataset, power was somewhat dependent on the presumed effective population size and time since separation, fluctuating from around 0.824 to 1 (Table 4). Nevertheless, putatively adaptive SNP loci dataset provided comparatively higher power than the all the SNP loci dataset, varying from 0.985 to 1 (Table 4). However, both the SNP loci datasets provided more than adequate power to detect genetic difference among the five broodstock populations of *P. monodon*.

### 3.3. Demographic Interpretations from F_ST_ Statistics and AMOVA Analysis

The pairwise F_ST_ value of five broodstock populations of *P. monodon* for the all SNP loci dataset were markedly lower than the pairwise F_ST_ estimates for the putatively adaptive SNP loci dataset (Table 5). For all the 194,259 SNP loci, pairwise F_ST_ estimates ranged from 0.003 to 0.153, with an overall average value of 0.089 (Table 5). For the 4983 putatively adaptive SNP loci, the pairwise F_ST_ value displayed higher values, ranging from 0.003 to 0.870, with an overall average value of 0.789. Low genetic differentiation, as observed by low pairwise F_ST_ value, was reported between the two wild-caught (MJ vs. MS) broodstock populations of *P. monodon* for all SNP loci (F_ST_ = 0.008; *p* = 0.001) and putatively adaptive SNP loci (F_ST_ = 0.106; *p* = 0.000) datasets. Similar pattern of genetic differentiation was also observed between the three domesticated (MMD vs. MMO; MT vs MMO; MT vs. MMD) broodstock populations of *P. monodon*, with pairwise F_ST_ values ranging from 0.003 (MMD vs. MMO) to 0.010 (MMO vs. MT) in all the SNP loci dataset, and 0.003 (MMD vs. MMO) to 0.042 (MMD vs. MT) in the putatively adaptive SNP loci dataset. In contrast, a high genetic divergence was observed between domesticated and wild populations, with pairwise F_ST_ values ranging from 0.145 (MMD vs. MS) to 0.153 (MJ vs. MT) in all the SNP loci dataset, and 0.836 (MJ vs. MMO) to 0.870 (MMD vs. MS) in the putatively adaptive SNP loci dataset. All of the pairwise F_ST_ comparison were significant (*p* ≤ 0.001), except for those between MMD and MMO for putatively adaptive SNP loci (*p* = 0.105) (Table 5).

When all SNPs were used, genetic variation inferred by a hierarchical AMOVA reveals the largest component of genetic variability (74.1%) within the individual level (Table 6). The portion of genetic divergence captured by AMOVA among the individuals was 16.1% (*p* = 0.000). A substantially low (9.8%) but significant divergence (*p* = 0.000) was observed among five broodstock populations of *P. monodon* (Table 6). Overall genetic structure yielded by AMOVA using all SNP loci resulted in a significant F_ST_ value of 0.098 (*p* = 0.000). Interestingly, hierarchical AMOVA based on the putatively adaptive loci revealed a high genetic divergence among five broodstock populations of *P. monodon* (80.8%; *p* = 0.000), but no differentiation was observed among individuals (0.0%; *p* = 0.483), while the remaining variation within individuals was 19.2% (Table 6). The hierarchical AMOVA for the putatively adaptive SNP loci also resulted in a significant F_ST_ value of 0.808 (*P* = 0.000), indicating a high level of genetic divergence of *P. monodon* broodstock populations. 

### 3.4. Genetic Structure Based on Clustering Analyses

**Figure 3:**
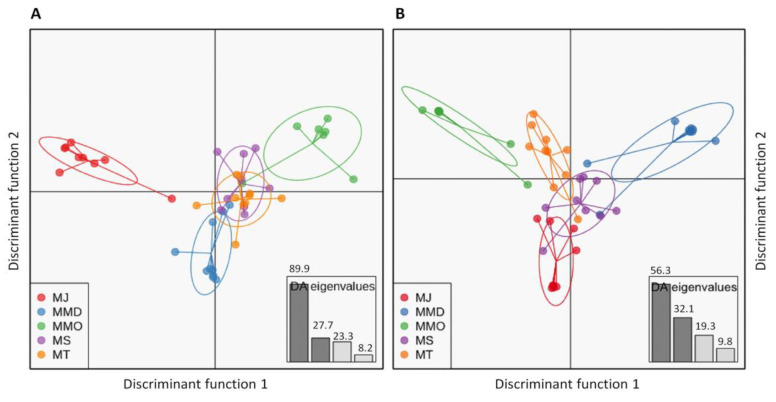
Plots showing the discriminant analysis of principal components (DAPCs) of genetic differentiation for the all (**A**) and the putatively adaptive (**B**) SNP loci of five broodstock populations of *Penaeus monodon*. Ovals are the inertial ellipse, dot represent individual genotypes and the line extends to centroids of each population Here, MJ indicate samples from Shizuoka, Japan (wild); MMD indicates samples from Mahajamba, Madagascar (Domesticated); MMO indicates samples from Hawaii, HI, USA (Domesticated); MS indicate samples from Setiu Wetland, Malaysia (wild); MT indicates samples from Petchaburi, Thailand (Domesticated).

**Figure 4:**
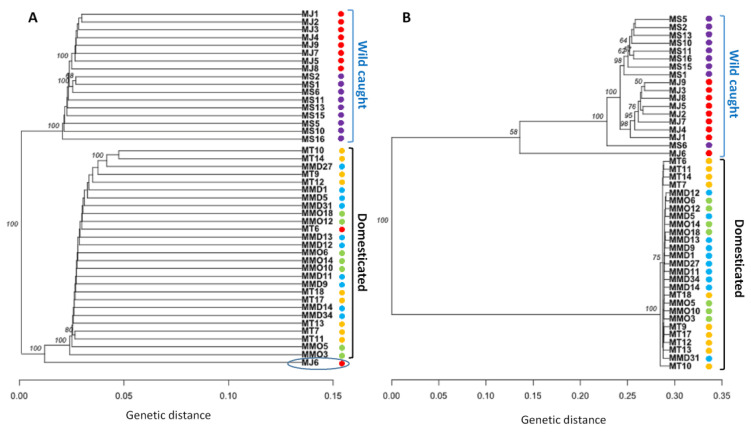
Neighbor-joining trees based on the Nei’s genetic distances for all SNP loci dataset (**A**) and the putatively adaptive panel of SNP loci dataset (**B**) of five broodstock populations of *Penaeus monodon* in the Indo-Pacific region. Branch nodes are denoted as the percentage of bootstrap support that was generated with 1000 replicates. Here, MJ indicate samples from Shizuoka, Japan (wild); MS indicate samples from Setiu Wetland, Malaysia (wild); MMD indicates samples collected from Mahajamba, Madagascar (Domesticated); MMO indicates samples from Hawaii, HI, USA (Domesticated); MT indicates samples from Petchaburi Province, Thailand (Domesticated). The numeric number next to the sample name abbreviation indicates the respective individual tag number during collection.

**Figure 5:**
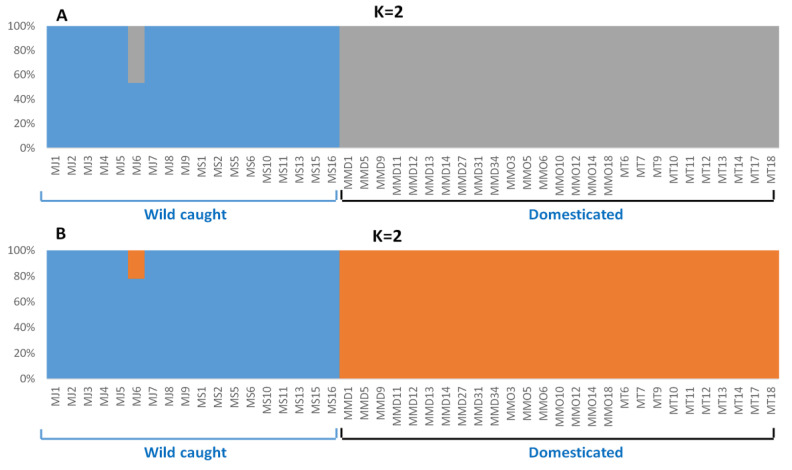
Bayesian STRUCTURE bar plot for all SNP loci dataset (**A**) and the putatively adaptive panel of SNP loci dataset identified by pcadapt approaches (**B**) of five broodstock populations of *Penaeus monodon* in the Indo-Pacific region. Each color represents the proportion of inferred ancestry from K ancestral populations and each bar represents an individual sample. Based on the delta K statistic, the best supported number of a posteriori genetic clusters was K = 2 for the standard admixture model. Here, MJ indicate samples from Shizuoka, Japan (wild); MS indicate samples from Setiu Wetland, Malaysia (wild); MMD indicates samples from Mahajamba, Madagascar (Domesticated); MMO indicates samples from Hawaii, HI, USA (Domesticated); MT indicates samples from Petchaburi Province, Thailand (Domesticated). The numeric number next to the sample name abbreviation indicates the respective individual tag number during collection.

Inferring from several spatial clustering analyses of all SNP loci and putatively adaptive SNP loci datasets, substantial differences in genetic structure patterns of five broodstock populations of *P. monodon* were observed. A discriminant analysis of principal components (DAPC) of all SNP loci datasets revealed that MT and MS populations were pronouncedly overlapped among each other, while others three populations were more or less discriminated from each other’s (Figure 3A). For putatively adaptive SNP loci dataset, DAPC analysis revealed that wild-caught broodstock populations (MS and MJ) were closely associated with each other’s (Figure 3B). The clustering patterns of all and putatively adaptive SNP loci datasets were also analyzed by neighbor-joining (NJ) trees based on Nei’s genetic distances, both of which revealed a divergent genetic structure of five broodstock populations of *P. monodon* into two clusters: (1) wild-caught populations (MS and MJ), and (2) domesticated populations (MMD, MMO and MT). However, NJ tree topology for all SNP loci dataset showed lower Nei’s genetic distance compared to the putatively adaptive SNP loci dataset, and only one wild-caught individual from Japan (MJ6) incongruently clustered with domesticated broodstock populations of *P. monodon* (Figure 4). Like NJ trees, STRUCTURE program also detects distinct genetic clusters (K) within a set of populations using a Bayesian clustering model. The number of clusters that best explain the genetic variation in the dataset is determined by estimating the cross-validation error. Similar to NJ trees, admixture analysis using Bayesian STRUCTURE of both all SNP loci and putatively adaptive SNP loci datasets also revealed two distinct genetic clusters (K = 2) within five broodstock populations of *P. monodon* (Figure 5). One spatial clustering was observed for wild-caught populations collected from MS and MJ regions, while another spatial clustering was observed for the domesticated populations collected from the MMD, MMO and MT regions (Figure 5).

### 3.5. GO Categorization of the Encoding Genes of Putatively Adaptive SNP Loci

**Figure 6:**
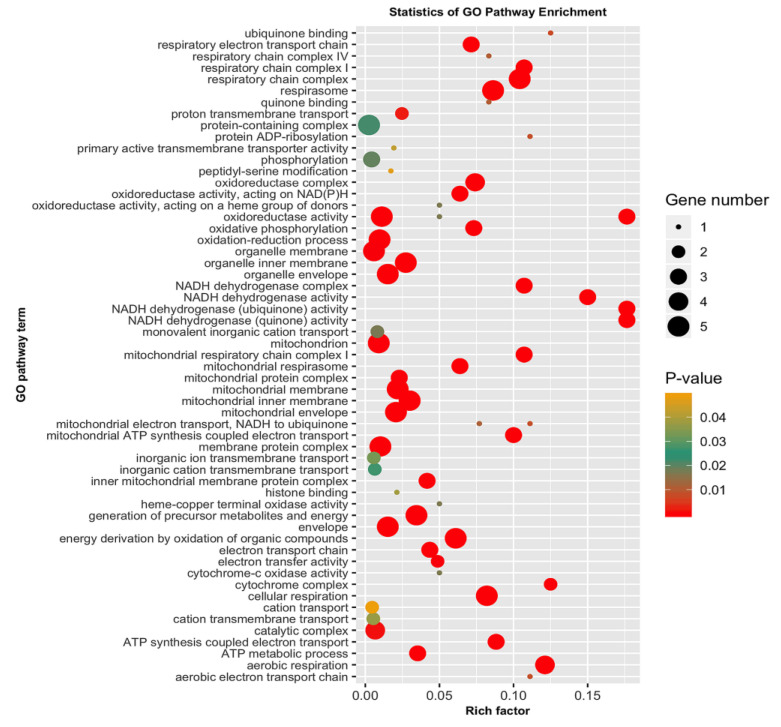
Gene ontology (GO) pathway enrichment analysis for the 50 genes encoded by the putatively adaptive SNP loci in *P. monodon* population.

**Figure 7:**
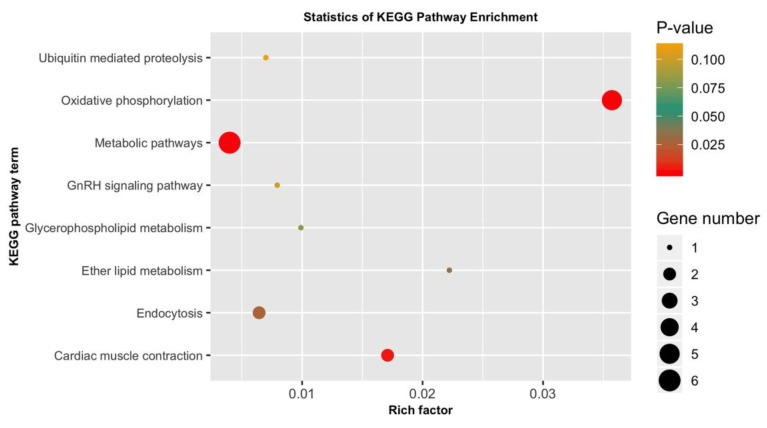
Kyoto Encyclopedia of Genes and Genomes (KEGG) pathways enrichment analysis for the 50 genes encoded by the putatively adaptive SNP loci in *P. monodon* population.

The 4983 putatively adaptive SNP loci sequences identified as outliers with the pcadapt approach were blasted against the genome of the Pacific white shrimp (*L. vannamei*), and 50 of them yielded significant matches with CDS amino acid sequences (Table S3). Among the 50 CDS amino acids sequences, 5 (COX3, ND4, ND6, CYTB, ND1) originated from mitochondrion, 16 were from known characterized protein, while the remaining 29 were uncharacterized protein Table S3. The 50 CDS were then subjected to GO and KEGG pathway enrichment analysis to discern the functional characterization of the genes encoded within the identified adaptive SNP loci. The GO results showed that most of the enriched pathways were linked to physiological functions, such as respiration, metabolism and energy derivation through mitochondrial oxidative phosphorylation and other organic compounds (Figure 6, Table S4). Many of these genes were involved in respiratory electron transport chain, respiratory chain complex, cellular respiration, aerobic respiration, respirasome, oxydoreductase activity, oxydative phosphorylation, organelle envelop, mitochondrion, mitochondrial envelope, membrane protein complex, generation of precursor metabolites and energy, energy derivation by oxidation of organic compounds, catalytic complex and the ATP metabolic process. KEGG pathway enrichment further demonstrated the involvement of the encoded genes in pathways related to metabolism, oxidative phosphorylation, cardiac muscle contraction and endocytosis, which is in coordination with the results of GO enrichment (Figure 7, Table S5). In addition, this study reveals an array of uncharacterized proteins with unknown functions. One uncharacterized protein LOC113820286 also appeared to be involved in respiration, the mitochondrial protein complex, the generation of precursor metabolites and energy and electron transport and ATP synthesis, like known mt proteins ND4, ND1, CYTB and COX3 (Table S4).

## 4. Discussion

Revealing the population structure patterns of *P. monodon* broodstocks is important for the systematic monitoring and management of both natural and wild populations. This study applied SkimSeq-based data to explore the variation of *P. monodon* broodstocks collected from natural habitats and shrimp farms in the Indo-Pacific regions. Loci with a high resolving power and potentially under selective process were detected. We observed a remarkable divergence between the domesticated and wild broodstocks. Although similar genetic discriminations were observed, putatively adaptive SNP loci most powerfully detected the genetic discrimination, as revealed by demographic interpretations and inferred clustering analyses. Compared to conventional molecular markers used for population genetic studies, our SkimSeq approach uses unbiased whole genome sequences to accurately identify the traces of selection that cause genetic differentiation using a lower coverage area with combination of small population size [77,78]. We noted that the impact of random genetic drift is larger in smaller sample size [79]. However, empirical studies have denoted that high throughput DNA sequencing have compensated smaller sample size with large number of generated SNP loci, to ensure high accuracy in estimating population genetic parameters [80,81,82]. Sample size as low as four individuals have been documented to be efficient in providing a precise estimate of FST values [83,84]. Indeed, a universal sample size rule may not be feasible to address the complexities in genomic kinship estimates [85]. As such, with the supporting power analysis output, the number of samples used in the present study is optimal in generating datasets of high precision.

AMOVA of putatively adaptive SNP loci showed a high total variance of 80.8% attributed to differences between the wild and domesticated groups. The low pairwise F_ST_ between wild populations of MS and MJ indicate that the two broodstock populations have genetic affinity, which could be the result of reciprocal transport, despite showing no significant geographic proximity, and this genetic pattern can be seen in other species, such as giant freshwater prawn (*Macrobrachium rosenbergii*) [86,87]. The NJ tree (Figure 4) and Bayesian STRUCTURE analysis (Figure 5) suggest limited genetic differentiation between all cultured populations (MMO, MMD and MT), implying that they might be derived from similar sources prior to domestication. Significant genetic similarity between the cultured populations may have stemmed from similar founder populations and selection procedures, which are normally practiced in shrimp industry.

Wild populations (MS, MJ) co-locate on another branch in NJ tree, indicating they have a separate origin from the cultured populations [86]. Besides, pairwise F_ST_ estimates, AMOVA, DAPC plot and STRUCTURE analysis agree with the genetic homogeneity of domesticated populations and their significant differentiation from the wild progenitor populations [88,89], particularly more significant for the putatively adaptive dataset. Significant genetic differentiation between domesticated stocks and wild populations has not been commonly observed in studies of the same species [37], but in other species such as salmon (*Salmo salar*) [90], grass carp (*Ctenopharyngodon idella*) [91,92] and Asian seabass (*Lates calcarifer*) [93].

In the present study, only one individual from MJ population was genetically identical to the cultured populations. Wild broodstocks, obtained as founders for shrimp domestication program, have been subjected to mass selection process over many generations, which may render lower genetic affinity between the cultured and wild populations. We also hypothesize that the founder broodstocks for the domestication program may have originated from more than one shrimp breeding company, and wild populations of different geographical localities. A lack of significant differentiation among domesticated populations may suggest the probability of a relatively short domestication history or genetically closely related populations [94].

Lack of gene flow between domesticated and wild populations is not unexpected when the confined environment of shrimp hatcheries and farms were taken into consideration. Moreover, our experimental design have defiled the potential occurrence of escapees from farms to the natural environment, given that all individuals were only obtained from populations, which were isolated completely from each other with a minimum of 500 km apart (MT and MS). The genetic structuring pattern being impacted by farm escapees was not uncommon, and has been reported in *P. monodon* [33,95]. However, more shrimp samples of wild and cultured stocks from different regions need to be analyzed to validate this genetic distinction. In fact, previous studies have suggested that fragmentation was commonly observed within the penaeid shrimp populations that were geographically separated by smaller distance [96].

The genetic homogeneity between the domesticated populations may be due to the artificial selection of favorable traits or adaptation to similar aquaculture practices. Aquaculture practices have been observed to reduce genetic variability in farmed reared stocks of other aquatic species [97,98,99]. Despite being geographically isolated, the genetic similarity among the wild populations, could possibly be linked to adaptive fitness to similar environmental conditions [100,101]. Convergent evolution has been documented in abalone (*Haliotis midae*) [102] and scallops (Pectinidae) [103], inhabiting analogous ecological niches, which subsequently develop consonant phenotypic traits. In addition, early population genetic studies on penaeid shrimp based on various techniques, including allozyme, RAPD and mtDNA analyses, showed that small genetic differences in these species were attributed to the dispersal ability, life history of shrimp and lack of physical barriers in the marine environment [32,95,104,105].

The effect of random genetic drift is more accentuated in smaller sample size [106]. Unequal sex ratio or differential reproductive contributions of the broodstocks in most breeding programs may cause random genetic drift [94,107]. In the present study, the low genetic differences between domesticated populations might be partially caused by random genetic drift or regionally different selective regimes [108]. Although this factor is a determinant key in dramatic depression of genetic variability in domesticated populations, it is beyond the scope of this study. 

Unique genes also persists in differentiating the domesticated and wild populations, which can be explained by selection of differentially favored alleles, holding particular reference to genetic improvement program in captive breeding environment [89]. The evidence of differential selection between the two groups (Figure 4 and Figure 5) highlighting the role of selection as a major evolutionary force in driving genetic divergence, specifically in domesticated populations. Selective pressures may be in part responsible for facilitating population variation; considering that these domesticated stocks have been undergoing grading procedures where undesired specimens were culled in the entire production system [89]. On the other hand, population heterogeneity connected to adaptation to environmental factors or ecological niches is well revealed in wild populations [102,109,110]. The development of ecotypes is well documented for many aquatic species in various environments where environmental clines persist [111,112,113]. 

The putatively adaptive SNP loci identified by the pcadapt approach also identifies genomic regions associated with the strong artificial selection during the domestication process over temporal scales. Of the 4983 putatively adaptive SNPs, only 50 genes were encoded successfully annotated through BLAST analysis. We have an array of uncharacterized proteins, with their functions are not known. Moreover, it was also observed that five mitochondrial genes (COX3, ND4, ND6, CYTB, ND1) were mutated among different populations of *P. monodon*. mtDNA has been widely used in population genetic studies to reconstruct phylogenetic relationships and analyze population structure [114], due to its unique characteristics such as maternal inheritance, neutrality, higher mutational rate than nuclear DNA and little to no recombination [115,116,117]. However, the prominent functions of a set of proteins encoded by mitochondrial genome in cellular energy production question its utility as a neutral marker [118]. In fact, our results from the KEGG pathway enrichment analysis has also suggested the presence of mutations under positive selection for the peptides, in which their functional properties are highly related in metabolic efficiency. Mitochondria produce 95% of cellular energy through oxidative phosphorylation of ADP (adenosine diphosphate) to form ATP (adenosine triphosphate). There is evidence that shows that several proteins containing mitochondrial encoded amino acids are involved not only in ion translocation, but also in mitochondrial respiration regulation [119,120]. Variation in mtDNA maybe taxon specific [121], and is linked to a range of environmental conditions affecting metabolic processes [122], development rates [123], biological ageing [124] and fitness. Mutations in the mitochondrial genome may affect the ability of mitochondria in ATP production [125], and putative maternal effects of mitochondrial genome on fish growth rate has been observed in other species, such as the Atlantic salmon [126]. Natural selection has also favored co-adaptative functions between mtDNA and nuclear DNA in maintaining optimal metabolic function and, thus, shaping the evolution of a species populations [127,128]. Despite being extensively utilized in manifold population structure studies, our findings have discovered the core functional properties of mitochondrial genome, particularly those involved in metabolic and energy productions as the driving force of population divergence. These fine attributes of mitochondrial functional properties warrant further new exploration and continual usage as one of the parameters in population genomics studies of various organisms. It has also been noticed that several genes are likely linked to other biological functions like molting (cuticle protein 21-like) [129], developmental process (neurotrophin 1-like) [130] and immunity (baculoviral IAP repeat-containing protein, serine protease 42-like) [131,132]. However, the present findings demonstrated that the nonsynonymous mutations in these encoded genes might be associated with different biological functions, which may enable *P. monodon* broodstocks to adapt to strong artificial selection during domestication process. 

## 5. Conclusions

We observed a pronounced genetic divergence between the wild and domesticated broodstock populations of *P. monodon* across the Indo-Pacific region. We suggested that similar founder populations, artificial selection regimes of desired commercial traits and local adaptive process to similar aquaculture practices have dramatically reduced the genetic heterogeneity of the domesticated stocks. Despite being geographically isolated, we denote that the patterns of genetic homogeneity in wild populations maybe strongly influenced by hydrographic conditions and ecological niches which are expected to increase the adaptive processes of the populations towards their natural habitats. Genetic differences between wild and domesticated populations presented in this study can be likely explained by a number of unique genes within the putatively adaptive SNP loci. These genes were found to be significantly associated with the mitochondrial genome. Our results from KEGG pathway analysis further reinforce the potential selective force which have diverged both groups. We noted the presence of polymorphism in the mitochondrial regions, notably those that are related to the energy production, metabolic functions, respiration regulation and developmental rates. The combination of these functional properties of peptide encoded by mitogenome are linked to various environmental parameters, and this likely acting to promote the genetic isolation of domesticated populations from their wild. Separated by geographical distance and various selection breeding programs, these two groups may each develop local adaptation to ecological niches, biological traits and demographic histories found within their thriving habitats. Taken together, this study has demonstrated the applicability of SkimSeq in a highly duplicated genome of penaeid shrimp, *P. monodon* specifically, across a range of genetic backgrounds and geographical distributions. This ultra-low sequencing coverage has enabled the sequencing of low number of individuals to achieve either similar or perhaps more powerful coverage, but with lower cost than other traditional GBS methods. In future work, larger sample sizes from a larger number of populations collected from wider distribution range should be included within a similar comparative framework, to validate if this specific trend represents a general rule associated with the genetic divergence of *P. monodon* broodstocks.

## Figures and Tables

**Table 1:** Summary of the sampling information of *Penaeus monodon* population collected from the different Indo-Pacific regions.

Locations	Location Abbreviation	Broodstock Source	Latitudes	Longitudes	Year
Mahajamba, Madagascar	MMD	Domesticated	16°02′52.8″	47°11′38.0″	2018
Hawaii, HI, USA	MMO	Domesticated	19°42′55.9″	156°02′34.6″	2018
Petchaburi Province, Thailand	MT	Domesticated	12°58′06.5″	99°37′48.0″	2019
Setiu Wetland, Malaysia	MS	Wild	5°40′38.3″	102°42′36.8″	2019
Shizuoka, Japan	MJ	Wild	34°56′25.9″	138°02′17.9″	2018

**Table 2:** Summary of the genome assembly, genome annotation, variants identifications and variant annotation results obtained from the skim sequencing (SkimSeq) data of *Penaeus monodon* broodstock population collected from different Indo-Pacific regions.

**Genome Assembly Statistics**	
Number of scaffolds	6,425,442
Size of largest scaffolds (bp)	15,300
Size of smallest scaffolds (bp)	100
Total scaffold size (bp)	1,531,786,734
Scaffold N50	306
**Genome annotation statistics**	
Number of Scaffolds with at least one protein sequence match	440,707
Number of Decapoda (order) protein sequences with match to scaffold	107,063
Number of SwissProt protein sequences with match to scaffold	42,351
**Variant identification results**	
Number of Scaffolds with at least one variant site	3,651,235
Total number of variants	17,226,908
**Variant annotation results**	
Total number of variants with annotations	1,347,070
Number of variants annotated with Decapoda proteins	1,339,785
Number of variants annotated with SwissProt proteins	142,710

**Table 3:** Number of single nucleotide polymorphism (SNP) loci remained after each quality filtering steps for dataset using all sampling groups.

Filtering Steps	Number of Loci
Total number of raw variants loci	17,226,908
Remaining SNP loci after excluded indels and MNP sites	13,530,393
Remaining SNP loci after excluded sites with MAF < 0.05	10,212,187
Remaining SNP loci after excluded sites with missing genotypes in >80% of the samples in any population	417,048
Remaining SNP loci after excluded sites with genotypes not in Hardy-Weinberg equilibrium in any population (PHWE < 0.001)	328,028
SNP loci remained after all quality filtering	194,259
Number of putatively adaptive SNP loci	4983

**Table 4:** Results of the power analysis conducted on all 194,259 SNP loci and 4983 putatively adaptive SNP loci.

All 194,259 SNP Loci			4983 Putatively Adaptive SNP Loci		
*t*	*Ne*	Power	*t*	*Ne*	Power
10	1000	0.945	10	1000	1.000
20	1000	1.000	20	1000	1.000
10	2000	0.884	10	2000	1.000
20	2000	0.912	20	2000	0.994
10	3000	0.824	10	3000	0.996
20	3000	0.902	20	3000	0.985

**Table 5:** Pairwise F_ST_ values (below diagonal) and associated *p* values (above diagonal) for the all SNPs loci and putatively adaptive SNP loci of giant tiger shrimp *Penaeus monodon* wild and domesticated broodstock populations collected from different Indo-Pacific regions.

	Wild		Domesticated		
	MJ	MS	MMO	MMD	MT
All SNP loci					
MJ	--	0.001	0.001	0.001	0.001
MS	0.008	--	0.001	0.001	0.001
MMO	0.151	0.147	--	0.023	0.001
MMD	0.150	0.145	0.003	--	0.001
MT	0.153	0.148	0.010	0.008	--
Putatively adaptive SNP loci					
MJ	--	0.000	0.000	0.000	0.000
MS	0.106	--	0.000	0.000	0.000
MMO	0.836	0.856	--	0.105	0.002
MMD	0.853	0.870	0.003	--	0.000
MT	0.850	0.868	0.035	0.042	--

**Table 6:** Molecular analysis of variance for the all SNP loci and putatively adaptive SNP loci of five giant tiger shrimp *Penaeus monodon* broodstock populations collected from different Indo-Pacific regions.

Source of Variation	Sum of Square	Variance Components	% of Variation	Statistics	*p*-Value
All SNP loci					
Within individuals	1,056,256	25,309.931	74.1	F_it = 0.259	-
Among individuals	1,286,226.1	5502.952	16.1	F_is = 0.179	0.000
Among populations	355,088.8	3342.094	9.8	F_st = 0.098	0.000
*Putatively adaptive SNP loci*					
Within individuals	10,205.5	238.184	19.2	F_it = 0.808	-
Among individuals	8192.9	−0.083	0.0	F_is = 0.000	0.483
Among populations	63029.16	1004.217	80.8	F_st = 0.808	0.000

## Supplementary Materials

The following are available online at https://www.mdpi.com/2079-7737/9/9/277/s1, Table S1: Number of read pairs before and after quality control of each individuals of *Penaeus monodon* collected from different Indo-Pacific region, Table S2: Reads alignment rate of each individual of *Penaeus monodon* collected from different Indo-Pacific region, Table S3: Summary of the gene annotation of the putatively adaptive panel of the SNP loci of broodstock populations of *Penaeus monodon* based on the reference genomes of Pacific white shrimp *Litopenaeus vannamei*, Table S4: Result of GO enrichment analysis showing significant GO pathway terms (*p* < 0.05) of the 50 genes encoded by the putatively adaptive panel of the SNP loci of *P. monodon* population, Table S5: Kyoto Encyclopedia of Genes and Genomes (KEGG) pathway terms and statistics of KEGG pathway enrichment analysis of the 50 genes encoded by the putatively adaptive panel of the SNP loci of *P. monodon* population.
