# Supplementary material for: Skim-Sequencing Based Genotyping Reveals Genetic Divergence of the Wild and Domesticated Population of Black Tiger Shrimp (Penaeus monodon) in the Indo-Pacific Region
Source: Biology (Basel). 2020 Sep 7;9(9):277. doi: 10.3390/biology9090277 (PMC7564732; doi:10.3390/biology9090277)
Supplement: Supplementary file 1 [file biology-09-00277-s001.pdf]

Supplementary Information

Table S1: Number of read pairs before and after quality control of each individuals of *Penaeus monodon* collected from different Indo-Pacific regions

Sample Name*	Total Sequencing Reads (Pairs)	Reads Not Matching to PhiX(Pairs)	Good Quality Reads (Pairs)	Good Quality Reads (%)
MJ1	7,501,746	7,501,746	5,302,812	70.69
MJ2	7,693,930	7,693,924	5,175,807	67.27
MJ3	7,535,617	7,535,616	5,532,492	73.42
MJ4	7,293,022	7,292,976	5,322,153	72.98
MJ5	7,631,038	7,631,038	5,715,376	74.90
MJ6	7,345,045	7,345,045	5,401,646	73.54
MJ7	7,580,049	7,580,049	5,593,183	73.79
MJ8	8,340,203	8,340,203	6,085,974	72.97
MJ9	7,753,121	7,753,121	5,717,979	73.75
MJ10	12,517	12,517	4,893	39.09
MMD1	7,792,584	7,792,584	5,332,622	68.43
MMD5	7,327,551	7,327,550	5,145,775	70.23
MMD9	8,158,674	8,158,674	5,804,675	71.15
MMD11	7,811,605	7,811,605	5,628,682	72.06
MMD12	8,053,964	8,053,964	5,937,327	73.72
MMD13	7,443,240	7,443,234	5,253,311	70.58
MMD14	7,749,584	7,749,584	5,623,374	72.56
MMD27	7,280,668	7,280,668	4,883,243	67.07
MMD31	7,088,953	7,088,953	5,005,280	70.61
MMD34	8,447,534	8,447,534	6,186,138	73.23
MMO1	8,361,743	8,361,743	6,053,904	72.40
MMO3	8,121,237	8,121,204	5,668,574	69.80
MMO5	8,357,962	8,357,962	6,031,317	72.16
MMO6	7,766,229	7,766,229	5,497,557	70.79
MMO7	8,416,611	8,416,611	6,123,625	72.76
MMO10	8,265,461	8,265,461	5,948,344	71.97
MMO12	7,792,509	7,792,509	5,440,718	69.82
MMO14	8,223,928	8,223,913	5,759,515	70.03
MMO16	8,148,675	8,148,675	5,533,838	67.91
MMO18	8,056,520	8,056,519	5,674,474	70.43
MS1	7,948,937	7,948,937	5,732,933	72.12
MS2	7,837,566	7,837,566	5,711,601	72.87
MS5	7,937,164	7,937,152	5,889,131	74.20
MS6	7,917,892	7,917,892	5,876,980	74.22
MS9	7,919,999	7,919,999	5,851,654	73.88
MS10	7,761,750	7,761,750	5,887,560	75.85
MS11	7,900,927	7,900,927	5,916,795	74.89

MS13	7,914,403	7,914,402	5,850,437	73.92
MS15	7,664,952	7,664,952	5,652,953	73.75
MS16	8,805,306	8,805,306	6,431,283	73.04
MT6	7,600,397	7,600,382	5,342,667	70.29
MT7	8,770,149	8,770,149	6,448,362	73.53
MT9	6,629,142	6,629,142	4,533,288	68.38
MT10	4,126,562	4,126,541	2,890,813	70.05
MT11	8,463,552	8,463,552	6,215,391	73.44
MT12	8,362,366	8,362,366	6,149,395	73.54
MT13	8,156,450	8,156,450	5,939,371	72.82
MT14	7,105,756	7,105,756	4,936,321	69.47
MT17	7,535,069	7,535,069	5,414,587	71.86
MT18	7,560,898	7,560,897	5,402,379	71.45

* MJ indicate samples collected from Shizuoka, Japan; MMD indicates samples collected from Mahajamba, Madagascar; MMO indicates samples collected from Hawaii, USA; MS indicate samples collected from Setiu Wetland, Malaysia; MT indicates samples collected from Petchaburi Province, Thailand.

Table S2. Reads alignment rate of each individual of *Penaeus monodon* collected from different Indo-Pacific regions

Sample Name*	Number of reads mapped	Percentage reads mapping (%)
MJ1	9,856,857	92.94
MJ2	9,540,691	92.17
MJ3	10,344,648	93.49
MJ4	9,944,631	93.43
MJ5	10,739,068	93.95
MJ6	10,069,684	93.21
MJ7	10,447,816	93.40
MJ8	11,300,492	92.84
MJ9	10,728,433	93.81
MJ10	8,531	87.18
MMD1	9,792,853	91.82
MMD5	9,590,602	93.19
MMD9	10,828,741	93.28
MMD11	10,413,745	92.51
MMD12	11,114,971	93.60
MMD13	9,692,815	92.25
MMD14	10,431,961	92.76
MMD27	9,108,367	93.26
MMD31	9,212,144	92.02
MMD34	11,594,319	93.71
MMO1	11,226,729	92.72
MMO3	10,498,031	92.60
MMO5	11,064,524	91.73
MMO6	10,115,968	92.00
MMO7	11,362,215	92.77
MMO10	10,951,391	92.05

MMO12	9,935,582	91.31
MMO14	10,609,452	92.10
MMO16	10,230,857	92.44
MMO18	10,335,406	91.07
MS1	10,737,455	93.65
MS2	10,668,590	93.39
MS5	11,026,208	93.61
MS6	11,029,103	93.83
MS9	11,040,427	94.34
MS10	11,021,484	93.60
MS11	11,141,403	94.15
MS13	10,973,053	93.78
MS15	10,618,867	93.92
MS16	12,032,928	93.55
MT6	9,744,112	91.19
MT7	11,983,408	92.92
MT9	8,314,184	91.70
MT10	5,381,318	93.08
MT11	11,622,375	93.50
MT12	11,560,469	94.00
MT13	11,046,514	92.99
MT14	9,217,054	93.36
MT17	10,070,579	92.99
MT18	10,070,023	93.20

* MJ indicate samples collected from Shizuoka, Japan; MMD indicates samples collected from Mahajamba, Madagascar; MMO indicates samples collected from Hawaii, USA; MS indicate samples collected from Setiu Wetland, Malaysia; MT indicates samples collected from Petchaburi Province, Thailand.

Table 3. Summary of the gene annotation of the putatively adaptive panel of the SNP loci of *Penaeus monodon* broodstock populations based on the reference genomes of Pacific white shrimp *Litopenaeus vannamei.*

GenBank Accession Number	Gene Description	Gene Symbol
XP_027210674.1	integrator complex subunit 10-like	LOC113804044
XP_027216986.1	cuticle protein 21-like	LOC113809542
XP_027218130.1	zinc finger protein 84-like	LOC113810690
XP_027219597.1	trichohyalin-like	LOC113811943
XP_027221865.1	zinc finger protein 91-like	LOC113813979
XP_027221929.1	crustacean hyperglycemic hormones-like	LOC113814051
XP_027222924.1	baculoviral IAP repeat-containing protein 7-like	LOC113815035
XP_027222958.1	serine protease 42-like	LOC113815063
XP_027223796.1	radical S-adenosyl methionine domain-containing protein 1, mitochondrial-like	LOC113815976
XP_027226535.1	semaphorin-1A-like	LOC113818546
XP_027230055.1	histone PARylation factor 1-like	LOC113821727
XP_027230443.1	golgin subfamily A member 6-like protein 2	LOC113822115
XP_027230799.1	ADP-ribosylation factor-like	LOC113822454
XP_027231314.1	neurotrophin 1-like	LOC113822960
XP_027234749.1	phospholipase D gamma 2-like	LOC113826071
XP_027239272.1	pro-resilin-like	LOC113830265
YP_001315037.1	cytochrome c oxidase subunit III	COX3
YP_001315040.1	NADH dehydrogenase subunit 4	ND4

YP_001315042.1	NADH dehydrogenase subunit 6	ND6
YP_001315043.1	cytochrome b	CYTB
YP_001315044.1	NADH dehydrogenase subunit 1	ND1
XP_027206737.1	uncharacterized protein LOC113800208	LOC113800208
XP_027206803.1	uncharacterized protein LOC113800258	LOC113800258
XP_027206995.1	uncharacterized protein LOC113800427	LOC113800427
XP_027207908.1	uncharacterized protein LOC113801282	LOC113801282
XP_027208800.1	uncharacterized protein LOC113802428	LOC113802428
XP_027211427.1	uncharacterized protein LOC113804734	LOC113804734
XP_027211978.1	uncharacterized protein LOC113805212	LOC113805212
XP_027212303.1	uncharacterized protein LOC113805499	LOC113805499
XP_027212477.1	uncharacterized protein LOC113805632	LOC113805632
XP_027215783.1	uncharacterized protein LOC113808555	LOC113808555
XP_027216240.1	uncharacterized protein LOC113808956	LOC113808956
XP_027216507.1	uncharacterized protein LOC113809181	LOC113809181
XP_027218523.1	uncharacterized protein LOC113811045	LOC113811045
XP_027218524.1	uncharacterized protein LOC113811046	LOC113811046
XP_027219801.1	uncharacterized protein LOC113812168	LOC113812168
XP_027224089.1	uncharacterized protein LOC113816249	LOC113816249
XP_027228409.1	uncharacterized protein LOC113820286	LOC113820286
XP_027230516.1	uncharacterized protein LOC113822177	LOC113822177
XP_027232687.1	uncharacterized protein LOC113824138	LOC113824138
XP_027233210.1	uncharacterized protein LOC113824649	LOC113824649
XP_027234007.1	uncharacterized protein LOC113825384	LOC113825384
XP_027234406.1	uncharacterized protein LOC113825773	LOC113825773
XP_027236530.1	uncharacterized protein LOC113827816	LOC113827816
XP_027237734.1	uncharacterized protein LOC113828876	LOC113828876
XP_027237905.1	uncharacterized protein LOC113829027	LOC113829027
XP_027239165.1	uncharacterized protein LOC113830159	LOC113830159
XP_027239512.1	uncharacterized protein LOC113830503	LOC113830503
XP_027239569.1	uncharacterized protein LOC113830563	LOC113830563
XP_027239571.1	uncharacterized protein LOC113830564	LOC113830564

Table S4. Result of GO enrichment analysis showing significant GO pathway terms (*P*<0.05) of the 50 genes encoded by putatively adaptive panel of the SNP loci of *P. monodon* populations

GO ID	GO Pathway Terms	P-value	Genes involved
GO:0098803	respiratory chain complex	6.59E-10	ND4, ND1, CYTB, COX3, LOC113820286
GO:0070469	Respirasome	1.61E-09	ND4, ND1, CYTB, COX3, LOC113820286
GO:0045333	cellular respiration	2.04E-09	ND4, ND1, CYTB, COX3, LOC113820286
GO:0015980	energy derivation by oxidation of organic compounds	8.33E-09	ND4, ND1, CYTB, COX3, LOC113820286
GO:0009060	aerobic respiration	2.23E-08	ND4, ND1, LOC113820286, COX3
GO:0006091	generation of precursor metabolites and energy	1.29E-07	ND4, ND1, CYTB, COX3, LOC113820286
GO:1990204	oxidoreductase complex	1.42E-07	ND4, ND1, LOC113820286, CYTB
GO:0005743	mitochondrial inner membrane	2.55E-07	ND4, ND1, CYTB, COX3, LOC113820286
GO:0019866	organelle inner membrane	3.96E-07	ND4, ND1, CYTB, COX3, LOC113820286
GO:0016655	oxidoreductase activity, acting on NAD(P)H, quinone or similar compound as acceptor	5.41E-07	ND4, ND1, LOC113820286
GO:0050136	NADH dehydrogenase (quinone) activity	5.41E-07	ND4, ND1, LOC113820286
GO:0008137	NADH dehydrogenase (ubiquinone) activity	5.41E-07	ND4, ND1, LOC113820286
GO:0003954	NADH dehydrogenase activity	8.40E-07	ND4, ND1, LOC113820286
GO:0031966	mitochondrial membrane	1.17E-06	ND4, ND1, CYTB, COX3, LOC113820286
GO:0005740	mitochondrial envelope	1.52E-06	ND4, ND1, CYTB, COX3, LOC113820286
GO:0045271	respiratory chain complex I	2.12E-06	ND4, ND1, LOC113820286
GO:0005747	mitochondrial respiratory chain complex I	2.12E-06	ND4, ND1, LOC113820286
GO:0030964	NADH dehydrogenase complex	2.12E-06	ND4, ND1, LOC113820286
GO:0042775	mitochondrial ATP synthesis coupled electron transport	2.57E-06	ND4, CYTB, COX3
GO:0042773	ATP synthesis coupled electron transport	3.66E-06	ND4, CYTB, COX3
GO:0006119	oxidative phosphorylation	6.21E-06	ND4, CYTB, COX3
GO:0022904	respiratory electron transport chain	6.65E-06	ND4, CYTB, COX3
GO:0031975	Envelope	6.72E-06	ND4, ND1, CYTB, COX3, LOC113820286
GO:0031967	organelle envelope	6.72E-06	ND4, ND1, CYTB, COX3, LOC113820286
GO:0005746	mitochondrial respirasome	9.16E-06	ND4, ND1, LOC113820286
GO:0016651	oxidoreductase activity, acting on NAD(P)H	9.16E-06	ND4, ND1, LOC113820286
GO:0022900	electron transport chain	2.75E-05	ND4, CYTB, COX3
GO:0016491	oxidoreductase activity	2.93E-05	ND4, ND1, CYTB, COX3, LOC113820286
GO:0098800	inner mitochondrial membrane protein complex	3.11E-05	ND4, ND1, LOC113820286
GO:0098796	membrane protein complex	4.42E-05	ND4, ND1, CYTB, COX3, LOC113820286
GO:0046034	ATP metabolic process	5.02E-05	ND4, CYTB, COX3
GO:0055114	oxidation-reduction process	5.79E-05	ND4, ND1, CYTB, COX3, LOC113820286
GO:0005739	Mitochondrion	7.65E-05	ND4, ND1, CYTB, COX3, LOC113820286
GO:0070069	cytochrome complex	9.69E-05	CYTB, COX3

GO:0098798	mitochondrial protein complex	0.00017513	ND4, ND1, LOC113820286
GO:0009055	electron transfer activity	0.00056502	CYTB, COX3
GO:0031090	organelle membrane	0.00057238	ND4, ND1, CYTB, COX3, LOC113820286
GO:1902494	catalytic complex	0.00135892	ND4, ND1, LOC113820286, CYTB
GO:1902600	proton transmembrane transport	0.00208719	ND4, COX3
GO:0048039	ubiquinone binding	0.00731775	ND4
GO:0006123	mitochondrial electron transport, cytochrome c to oxygen	0.00812766	COX3
GO:0006471	protein ADP-ribosylation	0.00812766	LOC113821727
GO:0019646	aerobic electron transport chain	0.00812766	COX3
GO:0048038	quinone binding	0.01055356	ND4
GO:0045277	respiratory chain complex IV	0.01055356	COX3
GO:0006120	mitochondrial electron transport, NADH to ubiquinone	0.01136093	ND4
GO:0016676	oxidoreductase activity, acting on a heme group of donors, oxygen as acceptor	0.01699483	COX3
GO:0004129	cytochrome-c oxidase activity	0.01699483	COX3
GO:0015002	heme-copper terminal oxidase activity	0.01699483	COX3
GO:0016675	oxidoreductase activity, acting on a heme group of donors	0.01699483	COX3
GO:0015672	monovalent inorganic cation transport	0.01743716	ND4, COX3
GO:0016310	Phosphorylation	0.01948521	ND4, CYTB, COX3
GO:0032991	protein-containing complex	0.02200883	ND4, ND1, CYTB, COX3, LOC113820286
GO:0098662	inorganic cation transmembrane transport	0.02703218	ND4, COX3
GO:0098660	inorganic ion transmembrane transport	0.03298345	ND4, COX3
GO:0098655	cation transmembrane transport	0.03703185	ND4, COX3
GO:0042393	histone binding	0.03843824	LOC113821727
GO:0015399	primary active transmembrane transporter activity	0.04235965	COX3
GO:0018209	peptidyl-serine modification	0.04704507	LOC113821727
GO:0006812	cation transport	0.04863327	ND4, COX3

Table 5. Kyoto Encyclopedia of Genes and Genomes (KEGG) pathway terms and statistics of KEGG pathway enrichment analysis of the 50 genes encoded by the putatively adaptive panel of the SNP loci of *P. monodon* populations.

ID	KEGG Pathway Terms	P-value	Genes involved
dre00190	Oxidative phosphorylation	1.09E-07	ND4, ND1, CYTB, COX3, LOC113820286
dre01100	Metabolic pathways	0.00112766	ND1, LOC113826071, LOC113820286, ND4, COX3, CYTB
dre04260	Cardiac muscle contraction	0.00422965	CYTB, COX3
dre04144	Endocytosis	0.02671585	LOC113822454, LOC113826071
dre00565	Ether lipid metabolism	0.03686536	LOC113826071
dre00564	Glycerophospholipid metabolism	0.07998574	LOC113826071
dre04912	GnRH signaling pathway	0.09863182	LOC113826071
dre04120	Ubiquitin mediated proteolysis	0.11110334	LOC113815035
